# Intracellular delivery of biologic therapeutics by bacterial secretion systems

**DOI:** 10.1017/erm.2017.7

**Published:** 2017-04-06

**Authors:** Barnabas James Walker, Guy-Bart V. Stan, Karen Marie Polizzi

**Affiliations:** 1 Department of Life Sciences, Imperial College London, London SW7 2AZ, UK; 2 Centre for Synthetic Biology and Innovation, Imperial College London, London SW7 2AZ, UK; 3 Department of Bioengineering, Imperial College London, London SW7 2AZ, UK

## Abstract

Biologics are a promising new class of drugs based on complex macromolecules such as proteins and nucleic acids. However, delivery of these macromolecules into the cytoplasm of target cells remains a significant challenge. Here we present one potential solution: bacterial nanomachines that have evolved over millions of years to efficiently deliver proteins and nucleic acids across cell membranes and between cells. In this review, we provide a brief overview of the different bacterial systems capable of direct delivery into the eukaryotic cytoplasm and the medical applications for which they are being investigated, along with a perspective on the future directions of this exciting field.

## Introduction to biologics

Recent years have seen an inexorable trend in the pharmaceutical industry away from ‘small-molecule’ drugs and towards the more complex macromolecular therapeutics known collectively as biologics. These include protein-based therapeutics—such as antibodies, hormones, growth factors and cytokines—and nucleic acid-based treatments such as short-interfering RNAs, DNA/RNA vaccines and gene therapies. The size and complexity of biologics provide the opportunity for a high level of specificity, allowing them to be extremely powerful but with fewer side-effects than traditional drugs. They can also make use of more powerful discovery tools such as rational design and directed evolution. As a result, biologics are coming to dominate the pharmaceutical industry, reportedly accounting for 40% of R&D funding (Ref. 1), 60% of patent applications amongst the top pharmaceutical companies (Ref. 2) and an impressive six out of the top ten highest grossing drugs in 2015 (Ref. 3).

Despite this, biologics present several significant challenges not faced by small-molecule drugs. Chief among these is the formulation and delivery strategy. Most small-molecule drugs are stable enough to survive being orally ingested and small enough to be absorbed into the blood through the gut lining and then diffuse across plasma membranes and into cells. In contrast, biologics are often highly susceptible to degradation in the stomach and intestinal tract and too large to be absorbed efficiently through the gut lining. Even when delivered intravenously, stability in the blood can be an issue and in particular, because of their size and charge characteristics, it remains extremely difficult for biologics to cross the plasma membrane and reach intracellular targets. As a result, most successful protein biologics have been limited to extracellular targets, which represent a tiny proportion of the potential targets in the body, whilst nucleic acid-based therapeutics, which can only act intracellularly, are yet to be widely adopted.

A significant amount of research has gone into a diverse range of solutions to the problem of intracellular delivery, including cell-penetrating peptides (Ref. 4), viral vectors (Ref. 5) and various polymeric, lipid and inorganic nanoparticle formulations (Refs 6, 7)—each with their own advantages and limitations.

However, another approach that is attracting attention from both the research community and the pharmaceutical industry is the use of engineered bacteria as a vector for drug delivery (Refs 8, 9, 10). As vectors, bacterial cells not only address manufacturing and stability difficulties by synthesising therapeutics on demand, but may also allow for unparalleled cell-type specificity and targeted delivery. In the future, it is hoped that their manufacturing and delivery capabilities will be coupled with their natural capacity for bio-sensing and signal integration to allow for more intelligent disease monitoring and dosage control (Ref. 11).

Furthermore, bacteria have evolved several highly specialised nanomachines, which allow them to deliver proteins and nucleic acids directly into the cytoplasm of target cells. In this review, we examine how these nanomachines may be exploited for direct cytoplasmic delivery of biologic therapeutics.

In the section ‘Bacterial secretion systems’, we provide a brief overview of the different bacterial secretion systems capable of facilitating direct cytoplasmic delivery. In the section ‘Applications’, we examine the different medical applications of these systems: reviewing the pre-clinical and proof-of-concept studies conducted so far. In the section ‘Design consideration’, we touch on several important design considerations for the application of bacterial secretion systems in a medical context. Finally, in the section ‘Clinical application’, we conclude with a discussion of future directions.

## Bacterial secretion systems

Bacterial secretion systems are currently classified into six major families known as the type I–VI secretion systems. Of these, only the type III, IV and VI systems have been shown to facilitate direct delivery into the cytoplasm of a target cell: with types I, II and V secreting only into the periplasm or extracellular space. To date, only the type III secretion system (T3SS) has been explored for medical applications; however, the different mechanisms and capabilities of the type IV system (T4SS) and VI secretion system (T6SS) may lend them advantages for certain applications in the future.

### Type III

The T3SS is arguably the most complex of the three—requiring over two dozen separate proteins for its functionality. However, it is also the most extensively studied because of its central role in the virulence of several important human pathogens such as *Escherichia coli, Salmonella, Vibrio, Pseudomonas, Shigella, Yersinia* and *Chlamydia*.

**Figure 1.**
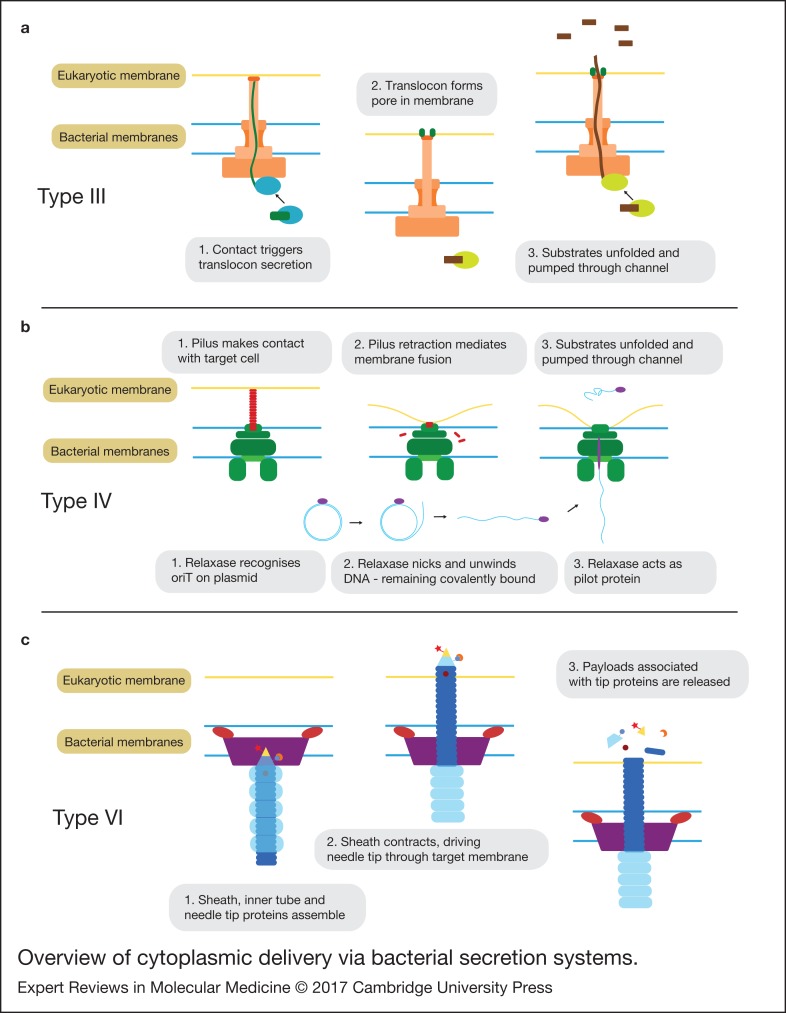
Overview of cytoplasmic delivery via bacterial secretion systems. Simplified schematics of the proposed mechanisms for type III, IV and VI secretion systems. Note: for the type IV only one of two suggested mechanisms is shown.

Structurally, the secretion complex is comprised a ‘basal body’ and a ‘needle-like’ filament giving it the appearance of a tiny ‘nano-syringe’ (Fig. 1a). Proteins are delivered by the T3SS in a two-step process. Firstly, contact with the appropriate target cell triggers the secretion of a hydrophobic ‘translocon’ protein, which inserts into the target membrane forming a pore. This creates a continuous path between the bacterial and eukaryotic cytoplasm through the lumen of the needle and pore. The dimensions of this channel require that the protein be unfolded during its passage. Although still poorly understood, substrate recruitment to the T3SS is thought to operate through the combination of an unstructured amphipathic *N*-terminal signal of approximately 15 amino acids along with a downstream chaperone binding site. The chaperone is thought to help maintain the protein in a partially unfolded state to facilitate delivery through the secretion channel. Both the protein unfolding and secretion are active processes, powered through a combination of ATPase activity and the proton motive force. Though there are still many unknowns surrounding the T3SS secretion mechanism, for an up-to-date and in-depth review we refer the reader to Notti and Stebbins (Ref. 12).

Delivery of heterologous protein substrates into human cells via the T3SS was first demonstrated using *Yersinia pseudotuberculosis* by creating a fusion of the heterologous protein (in this case adenylate cyclase) with the 50 N-terminal amino acids of the naturally injected effector YopE (Ref. 13). The technique has since been repeated many times and refined, using a variety of bacterial species and secretion signals, and has been explored for a number of different medical applications (see the section ‘Applications’).

Whilst the focus of this review is on intracellular delivery, it is of note that the T3SS is also capable of extracellular secretion. This capability has also been explored for medical applications in a number of proof-of-concept studies. For example, Chamekh et al. (Ref. 14) used the *Shigella* T3SS to deliver anti-inflammatory cytokines extracellularly in the gut in order to control inflammation. Another example is given by Shi et al. (Ref. 15), who used the T3SS of tumour targeting *Salmonella* to deliver angiogenic inhibitors into the tumour microenvironment in order to enhance the natural anti-tumour properties of the bacteria.

### Type IV

Type IV secretion systems (T4SSs) are the most wide-spread secretion system, present in both Gram-positive and Gram-negative species. This is largely because of their remarkable ability to deliver DNA as well as protein substrates, allowing them to facilitate horizontal gene transfer between bacteria (known as conjugation). However, like the T3SS, the T4SS is also used by several human pathogens, such as *Legionella pneumophila, Helicobacter pylori* and *Bartonella henselae*, to deliver protein toxins directly into human cells.

The T4SS machinery is formed of 13 proteins comprising: the core secretion apparatus, a pilus that facilitates contact with the target cell, and a coupling protein (T4CP) that recruits protein substrates to the secretion apparatus. The T4CP is also thought to provide power for substrate delivery through ATPase pump activity. The secretion signal for the T4SS is thought to reside in the 50 C-terminal amino acids, though some make use of additional targeting domains such as the Bartonella Intracellular Delivery (BID) domains of *Bartonella* species.

The details of the translocation process are still unclear. One theory is that depolymerisation of the pilus brings the membranes into close proximity allowing for a transient membrane fusion through which the substrates can be delivered (Fig. 1b). Another theory is that the pilus itself acts as a needle (much like in the T3SS), which directly penetrates the target membrane and through which the substrates travel in order to access the target cytoplasm (Ref. 16).

During bacterial conjugation, DNA is delivered by the T4SS as a single strand, covalently linked to a pilot protein called a relaxase. With the help of various accessory proteins, the relaxase recognises a specific sequence in the plasmid (the oriT), nicks it (whilst remaining covalently linked to the 5′ end) and unwinds the DNA with its helicase activity. Based on size considerations, it is thought that the relaxase (and other protein substrates), must be at least partially unfolded before secretion, though direct evidence is lacking. For more details, we refer the reader to the excellent review of Cabezon et al. (Ref. 16).

The only known example of naturally occurring, functional DNA transfer from bacteria into a eukaryotic cell is by the plant pathogen *Agrobacterium tumefaciens*, which uses a T4SS to deliver its ‘T-DNA’ into plant cells where it integrates into the genome and causes tumour formation. The high efficiency of this process along with the extremely broad host range has led to its wide-spread adoption in plant biotechnology for creating transgenic plants. *A. tumefaciens* has even been shown to be capable of transforming human cells (Ref. 17), albeit at very low efficiency and only with HeLa cells, which are notorious for their genetic promiscuity.

Interestingly, the protein-delivering T4SS of the human pathogen *B. henselae* has also been shown to be capable of delivering conjugative plasmids into human cells under laboratory conditions, presumably because of the high degree of homology with *Bartonella*’s conjugative apparatus, and the use of similar C-terminal secretion signals (Refs 18, 19). Whilst in both studies this transfer required only the expression of the conjugative relaxase and the presence of a plasmid with the corresponding oriT, the transfer efficiency was very low with this approach. Schröder et al. (Ref. 18) were able to increase the efficiency of the process 100-fold to ~2% by fusing the relaxase to a BID domain from a naturally-secreted *Bartonella* effector to help recruit it to the correct apparatus. Fernández-Gonzalez et al. (Ref. 19) were able to achieve a similar efficiency using a different conjugative plasmid by expressing both the relaxase and its native coupling protein (which presumably aided recruitment of the relaxase to the T4SS machinery).

However, these efficiencies are still low compared with some *A. tumefaciens* protocols, which can be as high as 90% (Ref. 20). This difference probably arises because the *A. tumefaciens* relaxase has evolved to facilitate nuclear entry and integration into the genome, whereas the Bartonella relaxase has not. Furthermore, *A. tumefaciens* co-delivers proteins to protect the DNA from degradation in the cytoplasm. Despite this, these results constitute a promising starting point for further development.

### Type VI

The T6SS is the most recently discovered and thus most poorly understood. The T6SS is also extremely wide-spread, with T6SS genes identified in around one-third of Gram-negative bacterial genomes (Ref. 21). It seems to be used primarily as a weapon to kill competing bacterial species by direct delivery of toxic payloads into the cytoplasm of target cells (Refs 22, 23). However, there are several known examples of T6SSs being used for virulence against eukaryotes (Refs 24, 25, 26, 27, 28).

The core secretion machinery consists of a sheath-like structure and an inner tube tipped with proteins that form a spike. Contraction of the sheath is thought to drive the inner tube into the target cell, puncturing the membrane. Payloads associated with the tip proteins, either covalently as fusion proteins or through noncovalent interactions, are then released into the cell (Fig. 1c). It is possible that some substrates are also loaded into the lumen of the tube and released upon depolymerisation (Ref. 23). The similarity of the T6SS structure and mechanism to the bacteriophage tail-spike has led many to speculate that the two may be evolutionarily related (Refs 22, 29).

Fusion of the beta lactamase enzyme either directly to the tip proteins (Ref. 27) or to other effectors associated with the tip (Ref. 30) has been used successfully as an assay for delivery into eukaryotic cells suggesting that similar fusions could support the delivery of other heterologous proteins. Whilst the carrying capacity and versatility of this approach for different substrates has yet to be explored, the ability to deliver substrates in native conformations may prove advantageous for proteins less amenable to unfolding and refolding. For example, Chen et al. (Ref. 31) found that extensive protein engineering was required in order to make certain Simian Immunodeficiency Virus (SIV) proteins suitable for secretion via the T3SS.

## Applications

The ability to deliver proteins and nucleic acids directly into the cytoplasm of human cells has an extremely wide range of potential applications in medicine. Here we distinguish between two broad classes of applications. In immunotherapy applications, the delivered proteins serve no functional purpose themselves; instead, they are partially degraded and the resulting epitopes displayed on the cell surface in order to modulate the immune system's response to the specific antigen delivered. In the second class of applications, which we refer to as ‘functional component delivery’, the protein itself performs some molecular function.

In this section, we review the progress made towards utilising bacterial secretion systems for these two broad classes of medical application. All the proof-of-concept and pre-clinical studies conducted so far have used the T3SS, which is the most well developed. However, it should be noted that, in principle, T4SS or T6SSs could be used towards similar ends in the future. At this stage, it is difficult to know which system may ultimately prove to be most appropriate for each application.

### Immunotherapy

One of the most promising and extensively explored applications of intracellular protein delivery via bacterial secretion systems is immunotherapy. Immunotherapy can be used either to stimulate an immune response against a particular antigen, as in vaccinations and cancer immunotherapy, or else to induce tolerance against allergens or disease associated auto-antigens.

In mammals, the adaptive immune response to antigens is controlled by two major antigen-presenting pathways. In the CD4^+^ branch, exogenous antigens, such as those on the surface of blood-borne pathogens, are engulfed by specialised dendritic cells, partially degraded in the lysosome, loaded into class II Major Histocompatibility Complexes (MHC-II), and displayed on the cell surface. Foreign antigens presented in MHC-II complexes are recognised by antigen-specific CD4^+^ helper T cells, which then go on to activate a specific immune response.

**Figure 2.**
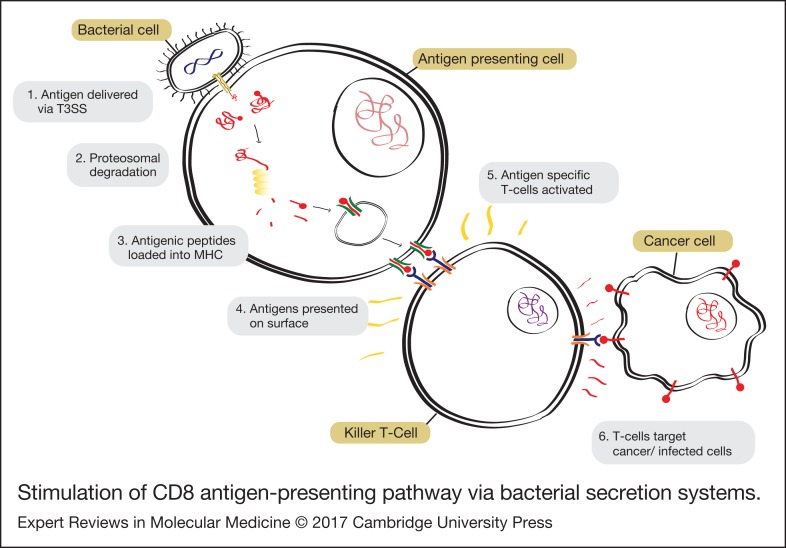
Stimulation of CD8 antigen-presenting pathway via bacterial secretion systems.

In the CD8^+^ branch, intracellular antigens, such as those from viruses or intracellular bacteria, are processed in the cytoplasm by the proteasome and loaded into class I Major Histocompatibility Complexes (MHC-I) that are then presented on the surface of the cell (Fig. 2). Foreign antigens presented in MHC-I complexes are recognised by antigen-specific CD8^+^ cytotoxic T cells, which can kill the infected cell and drive a global response.

Traditional vaccines are based on killed or attenuated pathogens, which are often able to stimulate an immune response similar to the wild pathogens. However, these can be slow and expensive to produce, purify and attenuate successfully—requiring extensive safety testing for each new vaccine. Modern biologic vaccines seek to eliminate much of these costs and speed up the development cycle by delivering only a few specific antigens from the pathogen in question—either as recombinantly produced and purified proteins, or the nucleic acids that code for them. The biologic approach can also be used to create vaccines against specific cancer markers, for which traditional methods are not applicable.

However, activation of the CD8^+^ T cell pathway is a particular challenge for biologic vaccines as it requires cytoplasmic delivery of the antigen or nucleic acids encoding it. Direct cytoplasmic delivery via bacterial secretion systems has been explored as one way to overcome this barrier and induce potent activation of the CD8^+^ branch of the adaptive immune system.

Delivering antigens this way could allow a universal bacterial chassis to be optimised for vaccination once and then reused for numerous different pathogens by changing only the antigens delivered. The use of a bacterial carrier has the additional advantage that they possess natural markers that activate innate immune receptors which can help to boost the adaptive response against the delivered antigen (Ref. 32). Furthermore, using a bacterial carrier can protect the antigen from degradation in the gut, allowing for oral delivery of the vaccine—a highly desirable property for ease of administration.

This concept has been explored for the treatment of infectious diseases, cancer and autoimmune diseases, each of which is discussed in more detail below. A comprehensive summary of the pre-clinical studies utilising the T3SS for immunotherapy is provided in Table 1.

**Table 1.** Summary of pre-clinical studies utilising the type III secretion system for vaccination/immunotherapy applications.

Carrier	Signal	Target	Antigen	Organism	Delivery	Outcome
*Salmonella typhimurium*	SptP (SPI1)	LCMV	Nucleoprotein epitope	Mice	Oral	Protection from lethal challenge (Ref. 33)
						Long lasting protective memory (Ref. 106)
	YopE (SPI1)	Listeria	LLO or p60	Mice	Oral	Protection from lethal challenge (Ref. 37)
	YopE (SPI1)	Listeria	LLO and p60	Mice	Oral	Protection from lethal challenge (Ref. 54)
	SspH2 (SPI2)	Listeria	LLO or p60	Mice	Oral	In vivo CD4^+^ and CD8^+^ T Cell priming (Ref. 73)
	SseF (SPI2)	Listeria	LLO	Mice	Oral	Highly reduced *Listeria* organ burden (Ref. 72)
	SopE (SPI1)	SIV	Gag	Rhesus Macaque	Oral	T-cell responses. No improvement in virus control (Ref. 40)
	SopE (SPI1)	Sarcoma	NY-ESO-1 (self)	Mice	Oral	Tumour regression (Ref. 41)
					Intratumoral	
	YopE (SPI1)	Fibrosarcoma	p60	Mice	Oral	Protection from tumour challenge (80% tumour free) (Ref. 42)
	SspH2 (SPI2)	Melanoma	HBx	Mice	Oral	Slowed tumour growth (Ref. 43)
	YopE (SPI1)	Fibrosarcoma	p60	Mice	Oral	50–52% complete tumour regression (Ref. 44)
					Intravenous	71–80% complete tumour regression (Ref. 44)
	SseF (SPI2)	Colon carcinoma, glioblastoma	Survivin (self)	Mice	Oral	Slowed tumour growth (prophylactic and therapeutic) (Ref. 45)
	SopE (SPI1)	Melanoma	TRP2 (self)	Mice	Oral	Prophylactic protection (6/8), tumour eradication (5/8) (Ref. 46)
	SseF (SPI2)	Melanoma	Survivin (self)	Mice	Oral	slowed tumour growth (Ref. 47)
	YopE (SPI1)	Melanoma	VEGF (self)	Mice	Oral	Prophylactic-reduced angiogenesis and tumour growth (Ref. 49)
	SseJ (SPI2)	Lymphoma Colon Carcinoma	Survivin (self)	Mice	Oral	Curative, long-lasting protective memory (Ref. 50)
*Yersinia enterocolitica*	YopE	Measles	Nucleoprotein capsid epitope	Mice	Oral	Protection (8/10) (Ref. 38)
	YopE	*Entamoeba histolytica*	Surface Lectin	Gerbils	Oral	Protection (7/10) or reduced load (Ref. 39)
*Yersinia pseudotuberculosis*	YopE	Listeria	LLO	Mice	Oral	Simultaneous CD4^+^ and CD8^+^ T Cell priming. Reduction in bacterial colonisation of spleen (Ref. 35)
*Pseudomonas aeruginosa*	ExoS	Melanoma	Ovalbumin	Mice	Subcutaneous injection	Prophylactic (7/8), curative (5/6) (Ref. 36)
						Prophylactic (5/6) (Ref. 51)
	ExoS	Melanoma	TRP2, Gp100 MUC18, Survivin (self)	Mice	Subcutaneous injection	Slowed tumour-related death (Ref. 52)
	ExoS	Glioblastoma	TRP2 and GP100	Mice	Subcutaneous injection	Slowed tumour-related death (Ref. 53)

#### Infectious disease

Use of a bacterial secretion system for vaccination was first tested by Russmann et al. (Ref. 33) who engineered *Salmonella typhimurium* to deliver specific epitopes of the lymphocytic choriomeningitis virus (LCMV) into mammalian cells via their T3SS. This was done by inserting short viral nucleoprotein epitopes between two domains of the naturally-secreted *Salmonella* effector SptP. After oral immunisation with this strain, mice were completely protected from a lethal dose of the virus. Importantly, the effects were only seen when the T3SS delivered the antigen directly into the cytoplasm—mutants in which the T3SS was limited to extracellular secretion did not provide the same protective effect.

The technique has since been repeated with a variety of carrier strains—such as *Yersinia enterocolitica* (Ref. 34), *Y. pseudotuberculosis* (Ref. 35) and *Pseudomonas aeruginosa* (Ref. 36)—and shown to have a similar protective effect against a wide range of pathogens, including *Listeria monocytogenes* (Ref. 37), the measles virus (Ref. 38) and *Entamoeba histolytica*—a protozoan parasite that causes dysentery and liver abscess (Ref. 39) (see Table 1 for a summary). Most studies have been conducted in mice or gerbil models with the exception of one trial, which tested a recombinant *S. typhimurium* for protection against SIV in rhesus macaques (Ref. 40). Whilst the treatment generated measurable CD8^+^ T-Cell responses, it did not provide protection against the virus—though it should be noted that SIV/HIV has been notoriously resistant to vaccination.

#### Cancer

More recently, there has been a shift towards applying this technique in order to stimulate the immune system against tumours as a potential cancer therapeutic, with most studies using either *S. typhimurium* or *P. aeruginosa* as a chassis (see Table 1 for a summary) (Refs 36, 41, 42, 43, 44, 45, 46, 47, 48, 49, 50, 51, 52, 53).

The reason for this shift away from infectious disease is unclear but likely reflects a greater need for effective cancer treatment options with fewer side-effects. Whereas killed or attenuated pathogens still represent the gold standard for infectious disease vaccines, there is no equivalent treatment for cancers.

Early studies used tumour models artificially expressing nonself antigens, such as p60 from *L. monocytogenes* (Refs 42, 44) or ovalbumin (Refs 36, 51). These pre-clinical studies in mice were often able to achieve protection against tumour challenge in a prophylactic setting, or even complete regression of existing tumours. Efficacy against real tumour associated antigens, such as survivin and vascular endothelial growth factor, has also been demonstrated, though often tumour growth could only be slowed. In order to improve performance, several groups have used adjuvants, such as ligands for activating Natural Killer T cells (Refs 45, 50). Another method used to improve the immune response is to deliver multiple antigens simultaneously with the same strain. This has been shown to improve efficacy against both tumour (Ref. 53) and infectious disease (Ref. 54) targets.

A limitation of this approach to cancer immunotherapy is that it requires tumour-specific antigens to be identified, which is often challenging. Nishikawa et al. (Ref. 41) explored a second approach to cancer immunotherapy that circumvents this issue. Instead of using antigen presentation in healthy cells to stimulate the immune system against tumour-associated antigens, this time the bacteria were injected directly into the tumour and programmed to deliver an immunogenic peptide directly into the tumour cells, which was then displayed on their surface. This allowed the immune system to recognise the tumour cells as foreign and kill them.

This strategy was able to produce significant tumour reductions in a mouse model. Remarkably, the treatment also resulted in an immune response to other natural epitopes enriched in the tumour cells—a process called epitope spreading. Because of this phenomenon, the bacteria do not have to deliver the antigen into every single tumour cell in order for it to be effectively cleared by the immune system. The authors identify that the delivery of the epitope to healthy cells could potentially lead to an autoimmune response, however during the period of the study no adverse effects of this nature were observed. This may have been be aided by *Salmonella*’s innate tumour-targeting abilities.

A problem with cancer immunotherapy is that the tumour microenvironment can be very immunosuppressive, with tumours producing many tolerogenic molecules to push T cells towards tolerance. Manuel et al. (Ref. 47) sought to overcome this by using two separate strains of *Salomonella*. One used the T3SS to deliver the tumour-associated antigen survivin, the other was programmed to lyse inside tumour cells, delivering a short-hairpin RNA targeted to knock down the tolerogenic gene Stat3, which normally drives expression of immunosuppressive molecules. They found that this combined treatment had a synergistic effect, suppressing tumour growth much more effectively than either strain on its own.

#### Autoimmune disease

Recently, the same group has exploited a similar technique to push the balance in the opposite direction, allowing them to promote tolerance to an auto-antigen in a model of type I diabetes (Ref. 55). Like before, a recombinant strain of *Salmonella* was used to deliver the auto-antigen into the cytoplasm via its T3SS. However, this time, in addition a separate strain was used to invade and lyse inside the cell delivering a plasmid encoding TGFβ—a cytokine known to promote tolerance. In a mouse model, a 3-week course of the combination therapy was able to prevent the development of diabetes in 75% of mice for the 25 weeks of the study with no signs of adverse effects.

This could prove a viable strategy for treating other auto-immune diseases, including type I diabetes, rheumatoid arthritis and multiple sclerosis, which together represent a significant health burden in developed economies. A similar strategy may also be applicable to the treatment of severe allergies.

### Functional component delivery

Although yet to reach the pre-clinical stage, recently, several groups have been exploring the use of the T3SS as a means of delivering functional proteins capable of performing specific tasks within the recipient cell. Whereas the inherent immunogenicity of bacteria is an advantage for immunotherapy applications, for delivering functional therapeutic proteins it poses a significant challenge. Provoking an immune response will not only result in the rapid clearance of the therapeutic bacteria, but may also result in undesirable and potentially dangerous side effects such as fever or inflammation. It is something that will have to be carefully monitored and controlled if bacteria are to be used safely and effectively as drug delivery vectors in vivo. As a result, functional protein delivery via bacterial secretion systems has been explored only in an ex vivo setting thus far. The complete range of functional proteins that could be delivered for therapeutic effect is too large to consider in its entirety here. Instead, we focus only on examples for which proof-of-concept studies have been performed.

#### Protein replacement

One application in which cytoplasmic delivery of a functional protein could have a therapeutic effect, is for replacing or supplementing the function of a natural protein that is mutated or underexpressed in disease. A proof-of-concept for this application was done by Polack et al. (Ref. 56) who used the T3SS of *P. aeruginosa* to replace a missing redox component into B-lymphocytes extracted from a patient with a form of chronic granulomatous disease. They were able to successfully restore NADPH oxidase activity in the cells (though the cells were not delivered back into the patient).

A limitation of this approach for the treatment of chronic diseases, compared with gene therapy for example, is the limited half-life of the delivered proteins. Maintaining the therapeutic effect would require regular treatment, extracting cells from patients and reapplying them is a highly invasive procedure.

Functional delivery of replacement proteins via bacteria in vivo may overcome this limitation, particularly if the vector was suitable for oral delivery. However, applications of this nature will require far greater control over the immunogenicity, toxicity and cell-type specificity of the bacterial chassis. Alternatively, in the future it may be possible to deliver the genes encoding the protein using the T4SS, either in an ex vivo or potentially even in an in vivo setting, allowing for a more prolonged therapeutic effect.

#### Cellular reprogramming

Another application of functional cytoplasmic protein delivery, for which half-life is less of an issue, is cellular reprogramming. Here, cells of one type, that is, skin cells or fat cells, are removed from the patient, converted into cells of a different type, such as pluripotent stem cells, and then reapplied to the patient. This form of cell therapy has huge potential for treating neurodegenerative disease, cardiovascular disease, muscular dystrophies and other forms of regenerative medicine.

Traditionally, this type of cellular reprogramming has been performed by transforming the cells with expression vectors for a cocktail of transcription factors (Ref. 57).

However, this can be a very low efficiency process and genomic insertion of the expression cassettes can lead to undesirable effects.

**Figure 3.**
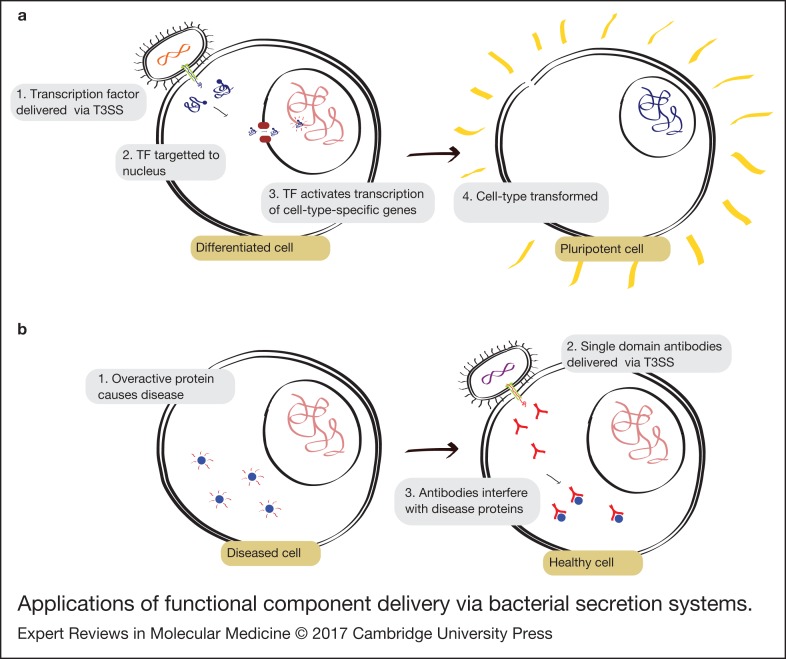
Applications of functional component delivery via bacterial secretion systems. (a) Illustration of cellular reprogramming via delivery of transcription factors by the T3SS. (b) Illustration of intracellular antibody delivery via the T3SS.

A protein-only approach could therefore be both more straightforward and safer, resulting in cells with no exogenous DNA. However, existing protein-only approaches suffer from degradation of the proteins during trafficking through the endomembrane system. Direct cytoplasmic delivery via a bacterial secretion system (Fig. 3a), could overcome this limitation providing a safe and efficient method of cellular reprogramming.

Steps towards this goal were first taken by Bichsel et al. (Ref. 58) who demonstrated that proteins delivered via the *P. aeruginosa* T3SS could be targeted to the nucleus by addition of a nuclear localisation tag. They then showed that the delivery of a MyoD fusion protein via the *P. aeruginosa* T3SS was sufficient to direct the differentiation of murine fibroblasts into myoblasts (Ref. 59) in vitro.

More recently, Berthoin et al. (Ref. 60) made fusions of the ExoS translocation sequence to canonical iPSC reprogramming transcription factors Oct4, Nanog and Sox2. They showed that each could be delivered into human fibroblasts independently via the *P. aeruginosa* T3SS. These modified transcription factors then localised to the nucleus and were able to efficiently activate elements of the pluripotency gene expression programme. The delivery strain could be fully cleared from the culture by the application of antibiotics, making the resultant cells safe for clinical use.

It is known that full, efficient reprogramming to a pluripotent state requires the combined action of multiple factors. It will be intriguing to see whether this can be achieved by simultaneous delivery of multiple reprogramming factors, either by a single strain or multiple strains. This could lead to a cheap, easy and efficient method for generating induced pluripotent cells.

#### Genome editing

Another form of cell therapy involves editing the genome of the extracted cells, for example to correct a genetic mutation (Ref. 61), reprogram T cells with specific cancer targeting receptors (Ref. 62), or edit cellular receptors to give HIV resistance (Ref. 63). Again, this process usually involves transformation of the cells with DNA cassettes expressing the gene editing machinery, i.e. CRISPR (Ref. 64), TALENs (Ref. 65) or zinc-finger nucleases (Ref. 66). Jia et al. (Ref. 67) used the *P. aeruginosa* T3SS, along with a nuclear localisation tag, to deliver TALENs into HeLa cells and perform targeted gene knockouts. They then extended this work to demonstrate single base-pair gene editing in human and mouse Embryonic Stem Cells as well as human induced Pluripotent Stem Cells—achieving improved efficiency over the traditional plasmid transfection method (Ref. 68).

In the future, this technique could also have potential as a method for cell-type specific in vivo gene editing. Whilst gene knockouts could be performed in vivo with a protein only approach, gene editing or gene insertion would require co-delivery of DNA —an application for which the T4SS may prove uniquely powerful.

#### Intracellular antibody delivery

Antibody therapies are currently by far the largest category of biologics, making up 30% of approved biologics between 2010 and 2014 and six of the top ten best sellers (Ref. 69). By exploiting the incredible power of the mammalian immune system, antibodies can be raised that will bind to almost any protein with extreme specificity. These antibodies can be used to target the immune system to attack specific cells or simply to interfere with the natural function of a protein (Fig. 3b). For example, the hugely successful drug Humira is an antibody which binds to TNFα, preventing it from triggering an inflammatory response.

Antibody therapies have thus far been limited to extracellular proteins. Being able to deliver them to the cytoplasm would open up a vast new space of potential targets, allowing specific interference with almost any cellular process (Fig. 3b). A significant step towards this goal was taken by Blanco-Toribio et al. (Ref. 70) who used the T3SS of enteropathogenic *E. coli* (EPEC) to deliver small single-domain antibodies, derived from camelids, into HeLa cells whilst retaining antigen-binding capabilities.

## Design considerations

In order to utilise bacterial secretion systems in a medical application there are several important design decisions to be made. Firstly, a starting strain, or ‘chassis’, with suitable secretion machinery must be chosen, often a single strain of bacteria will have multiple secretion machines that could be used and the most appropriate must be selected. Next comes the design of an appropriate fusion between the protein to be delivered and a signal peptide that will allow it to be secreted. Finally, the chassis may be modified in some way, for example via genome mutations, in order to optimise performance. In each of these choices attention must be paid to the effect on several major design criteria.

### Protein functionality

Most important is the functionality of the protein being delivered. Targeting a protein for secretion will usually require creating a fusion protein with a signal peptide from a naturally delivered effector and the choice of signal peptide may affect the folding/functionality of the protein being delivered. To help reduce these interference effects, the fusion protein can be designed such that the secretion tag is cleaved from the functional protein once inside the cell (Ref. 71). In immunotherapy applications, the nature of the fusion protein can also affect the efficiency with which an antigen is displayed. For example, both Hsp70 (Ref. 46) and the PADRE epitope (Refs 52, 53) have been shown to act as ‘immunochaperones’, facilitating presentation by MHC-I and II, respectively, when included in fusion with the antigen being delivered.

### Secretion efficiency

Another important factor is the secretion efficiency. This may vary considerably between different chassis and secretion machines and will also be affected by the signal peptide chosen, as different tags are often secreted at different levels. In addition, several genome mutations have been found to enhance secretion. For example, Russmann et al. (Ref. 35) found that mutations in the T3SS regulatory protein YopK in *Salmonella* increased secretion of the payload, whilst Epaulard et al. (Ref. 51) found enhanced secretion in a *P. aeruginosa* strain lacking the enzyme aroA for synthesising aromatic amino acids. However, beneficial mutations found in one strain cannot always be straightforwardly used in another: the beneficial aroA mutation found for *P. aeruginosa* had the opposite effect in *Salmonella* as it resulted in reduced expression of the fusion protein (Ref. 72).

### Timing and targeting

Just as important as the efficiency of secretion is when and where the payload is delivered. This is critical both for functional component delivery, where often a single diseased cell-type or tissue will need to be targeted, and also for immunotherapy, where the type of immune response generated depends heavily on the cell-types delivered. For example, Russmann et al. (Ref. 35) found that *Y. pseudotuberculosis* was able to induce simultaneous CD4^+^ and CD8^+^ T cell responses, whilst delivery of the same antigen by *Salmonella* resulted in only a CD8^+^ T cell response. This may be owing to differences in the life-cycle as *Salmonella* is primarily an intracellular pathogen, whilst *Yersinia* remains predominantly extracellular.

Not only do different strains of bacteria have different natural cell-type or tissue tropisms that can be exploited but different secretion apparatus within a single strain or even different substrates for the same apparatus can be expressed at different stages in the life-cycle. *Salmonella*, for example, possess two distinct T3SSs: *Salmonella* Pathogenicity Island 1 (SPI1) encodes the secretion apparatus and effectors necessary for invading the host cell, and is therefore expressed by extracellular bacteria early on in the invasion process and down-regulated subsequently, whereas Salmonella Pathogenicity Island 2 (SPI2) encodes the secretion apparatus and effectors for replication niche formation, and is only active once the bacterium is inside the cell. Panthel et al. (Ref. 73) found that some SPI2 signal peptides resulted in efficient CD4^+^ as well as CD8^+^ T cell activation, compared with SPI1 effectors which induced only CD8^+^ T Cells.

In the future, it may be possible to move beyond the natural tropisms of different bacteria and programmably control the cell-type specificity of the delivery strain. Recently, Pinero-Lambea et al. (Ref. 74) demonstrated that they could control the adhesion of *E. coli* to different cell-types by expressing cell-type-specific single-domain antibodies on their surface. This raises the tantalising possibility of being able to target-specific disease pathways in specific cell types, thereby minimising off-target effects even further and maximising efficacy.

### Immunogenicity and toxicity

Finally, a crucial factor to consider for any medical application is the toxicity and immunogenicity of the vector. Not only can high toxicity and immunogenicity lead to adverse side-effects, but they can cause a rapid clearing of the bacteria, limiting the therapeutic potential. On the other hand, for some immunotherapy applications, a residual degree of virulence can be important for generating the desired immune response and must therefore be carefully tuned. The toxicity and immunogenicity are largely determined by the choice of chassis and secondarily by various attenuating mutations. A common step for attenuating pathogenic secretion strains has been to knock out many of the naturally secreted effectors, many of which play a major role in virulence. This may have the added benefit of reducing competition for the secretion apparatus, thus enhancing secretion of the payload. The YopK mutant strain used by Russmann et al. (Ref. 35) was also found to have reduced toxicity, presumably because of a dis-regulation in the secretion of important virulence factors. Other general attenuating mutations, such as the aroA mutation used by Epaulard et al. (Ref. 51), can also decrease toxicity by reducing the replicative capacity.

In applications for which such attenuating mutations are insufficient, more extreme approaches can be taken. One such example is to use ‘killed-but-metabolically-active’ (KBMA) cells—a technique pioneered by Brockstedt et al. (Ref. 75). This involves knocking out the genes for nucleotide excision repair and using a cross-linking agent such as psoralen to cross-link the DNA. This prevents bacterial DNA replication without affecting metabolism and gene expression. Le Gouellec et al. (Ref. 76) demonstrated that KBMA *P. aeruginosa* could still deliver various antigens via its T3SS both in vitro and in vivo and found it to be highly effective but with reduced cytotoxicity. Another approach, used by Carleton et al. (Ref. 77), is to use nonreplicating bacterial mini-cells. Mini-cells are bacterial cells that lack chromosomal DNA altogether and are therefore unable to replicate. They can arise from aberrant cell division and can be produced in large quantities from bacterial strains with mutations in key cell division genes. Carleton et al. (Ref. 77) showed that mini-cells derived from T3SS-positive *S. typhimurium* were able to successfully prime antigen-specific CD8^+^ T cell responses in vivo in mice.

Several groups have recently begun tackling the toxicity problem from the other side: instead of attenuating secretion-competent but pathogenic bacteria, they seek to make nonpathogenic bacteria secretion-competent by transferring only the minimal secretion machinery, leaving behind all other virulence factors. Recently, T3SSs from *Vibrio parahaemolyticus* (Ref. 78), *Shigella* (Ref. 79) and EPEC (Ref. 80) have all been successfully expressed in nonpathogenic *E. coli* strains and shown to be capable of cytoplasmic delivery. The use of common laboratory strains of *E. coli* has the additional advantage that there exist far more well-characterised genetic parts and tools for use in *E. coli*. This may allow the utilisation of advances in the rapidly growing field of synthetic biology, which could see cytoplasmic delivery capabilities combined with existing detection modules (Ref. 81), logic circuits (Ref. 82), cell-type targeting (Ref. 74), and kill-switches (Ref. 83), leading to a more advanced and controllable therapeutic vector.

## Clinical application

The path from bench-to-bedside for live, engineered bacterial therapeutics is a long and winding one, with both technical and regulatory hurdles to overcome.

The use of bacteria for therapeutic purposes is not a recent phenomenon. It was not long after Louis Pasteur's famous experiments linking bacteria to disease in 1860 that live bacteria were first used for vaccination purposes in 1879; shortly after, in 1891, William Coley began experimenting with bacteria as a treatment for cancer (Ref. 84) and in 1917 a strain of ‘probiotic’ *E. coli* was first used as a treatment for Shigellosis (Ref. 85).

However, historical use has relied on the intrinsic therapeutic effects of natural bacterial species. Only recently has our ability to engineer bacteria progressed to the point where we can dream of tailoring a treatment to our specific needs. As such there are only a handful of clinical trials using engineered bacteria as therapeutics.

In general, these have fallen into three categories:

### Vaccine vectors

The engineering of vaccine vectors represents a natural progression from the previous practice of attenuation by random mutagenesis and selection, and, as such, the pathway to the clinic is the most established, with numerous clinical trials demonstrating safety and efficacy against both infectious disease (Refs 86, 87, 88, 89) and cancer (Refs 90, 91, 92, 93) targets. Engineered vaccines have benefited from an established regulatory framework and several such products are now in late-stage clinical development [see (Refs 10, 94) for more in-depth reviews].

### Tumour-targeting bacteria

Clinical application of tumour-targeting bacteria to treat cancer has, on the whole been less successful, with early trials demonstrating safety but with rapid clearance of the bacteria and poor efficacy (Refs 95, 96, 97, 98). The major challenge seems to be balancing the immunogenicity and toxicity with the efficacy. The poor efficacy is likely because of an over-attenuation of the strains. For an up-to-date review see (Ref. 8).

### Therapeutic gut microbes

The engineering of commensal bacteria for therapeutic delivery is a more recent phenomenon, with fewer trials. However, the use of nonpathogenic strains with a long record of safe use, such as *Lactobacillus* and *Bifidobacteria* often translates to a lower regulatory burden and several companies are beginning to take advantage of this. Intrexon currently has two clinical stage products in their ActoBiotics pipeline of engineered *L. lactis*, with promising initial results from a phase IB trial (Ref. 99). A second company, SynLogic, is following close behind with several products in the pre-clinical stage. For more information on the engineering of commensal bacteria we refer the interested reader to (Refs 100, 101).

As we have seen, intracellular delivery via the T3SS, T4SS and T6SS could add a powerful tool to all three of these application areas and therapies utilising this capability will presumably follow similar clinical trajectories depending on the application and chassis strain.

### Challenges

The dominant role of the immune system in any live bacterial therapeutic creates intrinsic difficulties in clinical translation because of significant differences in the immune systems of mice and humans, which makes it difficult to predict patient responses based on mouse studies (Ref. 102). Furthermore, there are often large differences in the immune responses of individual patients—for example the weakened immune responses of the elderly or those undergoing chemotherapy—which will affect treatment efficacy and safety.

In the future, it is envisaged that these difficulties could be addressed by implementation of feedback control systems within the therapeutic bacteria. Biomolecular implementation of such feedback loops could be realised, for example, through sensing of cytokine levels to gauge immune response and adjusting replication rate or expression of immunogenic factors accordingly. In the next generation of engineered bacterial therapeutics, biosensing and genetic logic will play an increasingly large role in improving the robustness of these therapies in the highly uncertain and changing environment of the human body.

However, another intrinsic problem with a live therapeutic is its ability to evolve, which, at best, leads to a loss of therapeutic effect and, at worst, can become harmful. It is likely that measures will have to be taken to increase the genetic stability of engineered bacterial therapeutics, as has been done for some laboratory strains (Ref. 103). Additional safety measures such as kill-switches (Ref. 83) or robust dependencies (Ref. 104) are also likely to be a feature for clinically applied engineered bacteria.

For a more comprehensive discussion on the promise and challenges of live-bacterial therapeutics we refer the interested reader to (Refs 8, 9, 10, 94, 105).

## Perspective

Complex macromolecules such as proteins and nucleic acids can act as highly specific therapeutics, with enormous potential for tackling disease. However, the delivery of macromolecules into the cytoplasm of specific cells is a highly challenging task. Bacteria have evolved several complex nanomachines, known as the T3SS, T4SS and T6SS, which are dedicated to this task, enabling high efficiency delivery of macromolecules directly into the cytoplasm of mammalian cells.

Thus far, only the T3SS has been explored for medical applications. However, the T4SS and T6SSs both possess unique features that may find important medical applications going forwards: the T4SS has the ability to deliver nucleic acids as well as proteins, which could potentially be exploited for gene therapy applications, whilst the T6SS may allow the delivery of proteins in native conformations.

Currently, the most well-developed medical application for cytoplasmic delivery by the T3SS is immunotherapy, which turns the inherent immunogenicity of the bacterial chassis into an advantage. Whilst examples are yet to reach the clinic, the work on the *P. aeruginosa* chassis has spawned a spin-out company (APCure), which is looking to commercialise the technology.

More recently, several groups have begun exploring other applications of direct cytoplasmic protein delivery, including protein replacement, cellular reprogramming, genome editing and antibody delivery. For these applications, the immunogenicity of the chassis is a potentially limiting factor and, in the short term at least, in vivo applications are likely to be restricted either to mucosal surfaces such as the gut, where immunogenicity is less of an issue, or the tumour microenvironment, where the immune system is often suppressed.

Several applications, such as cellular reprogramming and genome editing, may be applicable in an ex vivo setting, where immunogenicity is also less of an issue. However, in this setting, the technique will face fierce competition from more aggressive chemical and physical techniques that are not applicable in vivo. The unique advantages of a bacterial vector, such as oral delivery, on-site manufacturing, cell-type targeting, biosensing and signal processing are fully realised only in an in vivo setting.

Looking forward, there is still much to be learned about the functionality of all three secretion systems. However, we are now at a point where we will begin to see heterologous delivery via the T4SS and T6SS being developed and explored for medical applications—following in the footsteps of the type III. As we enter the era of synthetic biology, it is anticipated that the knowledge gained over the past two decades on the function of these secretion systems and their application will begin to be combined with other efforts to make bacterial vectors more controllable, so that they may finally make the leap from the lab bench into the clinic.
